# Transfusion Burden in Allogeneic Hematopoietic Stem Cell Transplantation over Time: Experience from a Single Institution

**DOI:** 10.3390/jcm12103467

**Published:** 2023-05-15

**Authors:** Pilar Solves, Javier Marco-Ayala, Miguel Ángel Sanz, Inés Gómez-Seguí, Aitana Balaguer-Roselló, Ana Facal, Marta Villalba, Juan Montoro, Guillermo Sanz, Javier de la Rubia, Jaime Sanz

**Affiliations:** 1 Haematology Department, Hospital Universitari i Politècnic La Fe, 46026 Valencia, Spain; 2 Centro de Investigación Biomédica en red Cancer, Instituto Carlos III, 28029 Madrid, Spain; 3 Haematology Department, University Hospital “Morales Meseguer”, 30007 Murcia, Spain; 4 School of Medicine and Dentistry, Catholic University of Valencia, 46010 Valencia, Spain

**Keywords:** blood transfusion, hematopoietic stem cell transplantation, transfusion independence

## Abstract

Introduction: Transfusion plays a main role in supportive treatment for patients who receive an allogeneic hematopoietic stem cell transplantation (HSCT). In this study, we compare the transfusion requirements of patients undergoing different modalities of HSCT according to different time periods. The objective is to assess the evolution of HSCT transfusion requirements over time, from a single institution. Methods: The clinical charts and transfusion records of patients who underwent HSCT of different modalities at La Fe University Hospital during a twelve-year period were reviewed (2009–2020). For analysis, we divided the overall time into three periods: 1 from 2009 to 2012, 2 from 2013 to 2016 and 3 from 2017 to 2020. The study included 855 consecutive adult HSCT: 358 HLA-matched related donors (MRD), 134 HLA-matched unrelated donors (MUD), 223 umbilical cord blood transplantation (UCBT) and 140 haploidentical transplants (Haplo-HSCT). Results: There were no significant differences in RBC and PLT requirements or transfusion independence among the three time periods for MUD and Haplo-HSCT. However, the transfusion burden increased significantly for MRD HSCT during the 2017–2020 period. Conclusion: despite HSCT modalities having evolved and changed over time, overall transfusion requirements have not significantly decreased and continue to be a cornerstone of transplantation-supportive care.

## 1. Introduction

Blood product transfusion plays a main role in the supportive care of patients who receive an allogeneic hematopoietic stem cell transplantation (HSCT) [1,2]. Red blood cell (RBC) and platelet (PLT) transfusions are needed for prolonged aplasia periods that patients suffer along with the HSCT procedure. Once engraftment occurs, some patients continue to be transfused due to the unexpected and expected complications arising from the graft–host interactions and the immunosuppression status [3,4].

Blood banks must assure adequate transfusion management for each moment of transplantation, paying special attention to the ABO group mismatch between donor and recipient [5]. Since transfusion thresholds are not universally established for RBC or PLT, transfusion practices are highly variable among centres [6]. In addition, patients who receive an HSCT are among the highest platelet transfusion consumers, not only due to the transplantation but to the primary disease as well [7]. Despite its obvious benefits, blood product transfusion has been related to adverse effects in the HSCT setting [8]. Thus, transplantation units should assess the transfusion burden of the procedure.

In this study, we aimed to assess the evolution of HSCT transfusion requirements over time. For this purpose, we compared the transfusion requirements of patients undergoing different modalities of HSCT according to different periods at our hospital.

## 2. Material and Methods

### 2.1. Patient Characteristics and Transplantation Modalities

We reviewed the clinical charts and transfusion records of patients who underwent HSCT of different modalities at La Fe University Hospital during a twelve-year period (2009–2020). For the analysis, we divided the overall time into three periods: 1 from 2009 to 2012, 2 from 2013 to 2016 and 3 from 2017 to 2020, respectively. The study included 855 consecutive adult HSCT: 358 HLA-matched related donors (MRD), 134 HLA-matched unrelated donors (MUD), 223 umbilical cord blood transplantations (UCBT) and 140 Haploidentical (Haplo-HSCT). HLA typing was determined at 10 loci (A, B, C, DRB1, DQB1). MRD and MUD were defined as HLA matched (10/10) or mismatched at 1 locus (9/10). Umbilical cord blood units were required to be HLA-matched at ≥4/6 loci. HSCT procedures including conditioning regimes, graft versus host disease (GVHD) prophylaxis and supportive care have been previously reported by our group [9,10,11,12]. The conditioning regime consisted of either a conventional myeloablative regime (MAC) or reduced-intensity conditioning (RIC) for patients unfit to receive MAC. The intensity of the conditioning regime depended on the disease status at transplant, performance status, and comorbidities of the patient, according to institutional protocols. Post-transplantation cyclophosphamide (PT-Cy) was used as GVHD prophylaxis in Haplo-HSCT and since 2017 it has also been used in MRD and MUD transplantations [11].

### 2.2. Transfusion Protocol

The study considered the transfusion burden (number of RBC units and platelet concentrates) during the first 90 days of allogeneic transplantation starting from the day of transplantation. All blood products were leukocyte-filtered and irradiated with 25 Gy. Prophylactic PLT were transfused when platelet counts were <20 × 10^9^/L for UCBT and <10 × 10^9^/L for the rest of HSCT. Whole-blood pooled random platelets (PLT) were mostly transfused, unless patients developed refractoriness. Red blood cell (RBC) concentrates were transfused when the hemoglobin was <80 g/L or symptoms of anaemia were present. The transfusion guidelines evolved from 2009 through 2020. Traditionally 2 RBCs were transfused in each episode and in 2019 the transfusion policy changed to 1 RBC per episode.

The ABO type of RBC and PLT transfused after the HSCT was determined according to the donor and recipient blood groups, as described previously [13]. For minor ABO-incompatible transplantations, donor-type RBCs and recipient-type PLTs were transfused, while for major ABO-incompatible recipients, recipient-type RBCs and donor-type PLTs were transfused. For bidirectional ABO-incompatible patients, group O RBCs and group AB platelets were scheduled.

PLT transfusion independence was defined as the last day of PLT transfusion, with no PLT transfusions in the following 7 days. RBC transfusion independence was defined as the day of the last transfusion, with no RBC transfusion in the following 30 days. PLT and RBC transfusion requirements at 90 after UCBT were recorded.

### 2.3. Statistical Analysis

Descriptive statistics are presented for variables. Results are expressed as median and range for continuous variables and as numbers with percentages for categorical variables. The Kolmogorov–Smirnov test was used to check the normal distribution of the variables.

The primary endpoints were the number of RBC and PLT units transfused, and time to RBC and PLT transfusion independence according to the time period and HSCT modality.

Categorical variables were compared using the Chi-square test or the Fisher exact test. The Mann–Whitney U test or the Kruskal–Wallis test for continuous variables was used to compare the groups when applicable. The cumulative incidence of RBC and PLT transfusion independence and transplant-related mortality was calculated in a competing risk setting. Overall, survival was calculated using the Kaplan–Meier method. All *p* values reported were two-sided and *p* value < 0.05 was considered statistically significant. Statistical analysis was conducted using EZR version 1.54 (22), a graphic user interface for R (The R Foundation for Statistical Computing) and SPSS (version 15, SPSS Inc., Chicago, IL, USA) [14].

## 3. Results

### 3.1. Patient and Transplantation Characteristics

Table 1 shows the characteristics of all patients undergoing different HSCT platforms according to the three studied periods. A total of 257, 297 and 301 HSCT of different platforms were performed during period 1, 2 and 3, respectively. The most common disease type was acute leukaemia. The stem cell source was mostly peripheral blood and umbilical cord blood. During period 3, patients undergoing HSCT were significantly older and received a graft with higher CD34+ cell content. More UCBT were performed during period 1 while more MUD and haploidentical transplantations were performed during period 3. PT-Cy + methotrexate + sirolimus was the most used combination to prevent graft versus host disease in the last period. The median number of days until reaching neutrophil > 0.5 × 10^9^/L and platelets > 20 × 10^9^/L, were higher for patients who received HSCT during the last analysed period.

### 3.2. Transfusion Outcome

When analysing all 855 HSCT, the median of RBC transfused at 30 days and 90 days after transplantation were, respectively, 4 units and 5 units for all three analysed periods (*p* = 0.82 and *p* = 0.9, respectively). There were not significant differences either in PLT transfusion at 30 days (median 10, 8 and 9 units in periods 1, 2 and 3 respectively; *p* = 0.11) and 90 days after transplantation (median 8, 7 and 8 units; *p* = 0.42).

**Figure 1:**
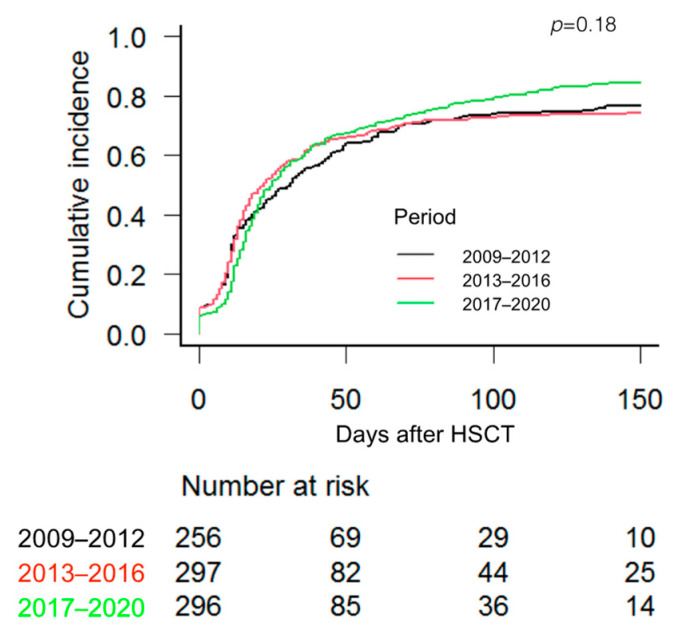
Cumulative incidence of red blood cell transfusion independence according to the 3 studied periods. Cumulative incidence of competing events and Gray test were used.

**Figure 2:**
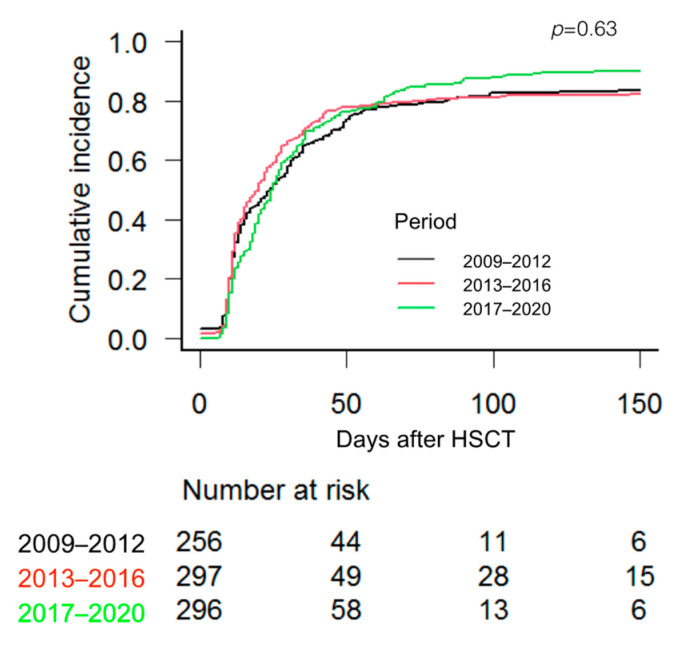
Cumulative incidence of platelet transfusion independence according to the three periods. Cumulative incidence of competing events and Gray test were used.

The median time to reach RBC transfusion independence was 17.5 days, 14.5 and 21 days in patients who received an HSCT during the time periods 1, 2 and 3, respectively (*p* = 0.187), while the median time to reach PLT transfusion independence was 16, 15 and 23 days for time periods 1, 2 and 3, respectively (*p =* 0.632). Figure 1 and Figure 2 show the cumulative incidence of RBC and PLT transfusion independence according to the 3 periods.

Transfusion requirements and transfusion independence for each HSCT modality according to the 3 periods are shown in Table 2. There were no significant differences in RBC and PLT requirements or transfusion independence among the 3 time periods for MUD and Haplo-HSCT. However, the transfusion burden in the first 30 days after transplantation increased significantly for MRD HSCT during the 2017–2020 period.

In the MRD setting, platelet transfusion independence was delayed in patients who received PT-Cy compared to other GVHD prophylaxis (median 20 vs. 11 days; *p* = 0.01). We also found a trend towards longer RBC independence (20 vs. 12 days; *p* = 0.07). However, PT-Cy did not show significant differences in transfusion independence in the MUD setting (median 26 vs. 13 days for RBC transfusion independence, *p* = 0.5; 25 vs. 12.5 days for platelet transfusion independence, *p* = 0.2).

**Figure 3:**
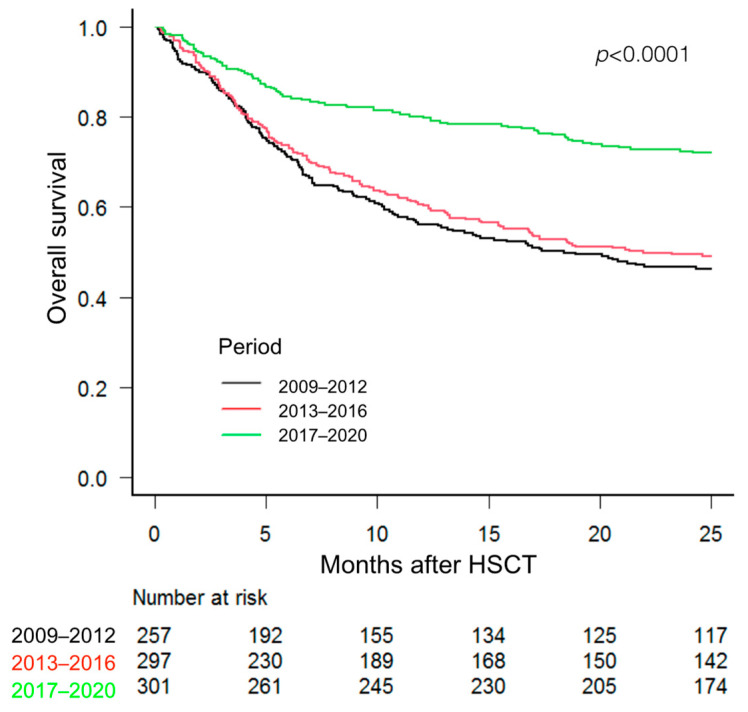
Overall survival of patients undergoing HSCT according to the 3 study periods. The Kaplan–Meier test was used.

Figure 3 shows the overall survival according to the three studied periods Survival was significantly higher for patients who received an HSCT during the last period (2017–2020).

## 4. Discussion

This is a large study focused on the transfusion requirements of HSCT from a single institution over time. We included all the consecutive HSCT performed on 855 patients over a 12-year period. The results show an overview of how HSCT practices and transfusion requirements have evolved with time in our tertiary-care hospital. For the first period analysed (2009–2012), our centre performed almost as many UCBT as MRD HSCT, while during the third period (2017–2020) UCBT have been mostly replaced by MUD and haplo-HSCT. As expected, MRD HSCT has remained stable since the HLA-matched related donor is the standard approach when available.

When performing an overall analysis of all transplantations, the transfusion requirements and transfusion independence for RBC and PLT were not significantly different over time, despite the important differences in transplantation platforms used in each period. This may be due, at least in part, to the increase in transfusion requirements in PT-Cy-based GVHD prophylaxis that is compensating for the lower rate of UCBT in the most recent period.

However, when the transfusion outcome is analysed for each HSCT modality, there are some relevant changes over time in patients undergoing UCBT and MRD. Among transplantations, the UCBT platform is clearly the highest blood product consumer [15,16,17]. UCBT is characterized by the low cell content of the stem cell graft that delays the myeloid and lymphoid engraftment and increases the transfusion needs [17]. However, a trend of fewer transfusion requirements is observed over the years in UCBT, although without reaching any statistical significance. The focus must be that UCBT is represented in different proportions along the analysed time. A better knowledge of the engraftment kinetics and the overall procedures could have contributed to the lower transfusion trend.

One of the most important findings of this study is the increase in transfusion needs at day +30 and delayed platelet and RBC transfusion independence for patients who received MRD transplantations during the most recent studied period (2017–2020) as compared to the previous time. MRD is the transplantation modality that has the lowest transfusion requirements [18,19,20,21]. The median number of units transfused is 4 RBC and 4 PLT by day 30 of transplantation [18]. It would be reasonable to hypothesize that the best knowledge of the procedure and the long experience gained over the years could contribute to reducing the transfusion burden. On the contrary, our group has recently found that the addition of PT-Cy in the graft versus host disease prophylaxis causes a delay in the myeloid engraftment, and multivariable analysis confirmed the significant impact of PT-Cy on this event. According to our previous study, myeloid engraftment is delayed by 3 days of median as compared to MRD transplantation without PT-Cy. In addition, we found a clear impact of the GVHD prophylaxis scheme including cyclophosphamide in the increased transfusion burden and delayed transfusion independence of patients receiving a MRD [22]. A delay in myeloid engraftment and a higher incidence of haemorrhagic cystitis are the proposed underlying mechanisms. As shown in the present results, most patients during the last studied period received a prophylaxis scheme including PT-Cy. The impact of PT-Cy in other transplantation modalities such as MUD seems to be less important, although it is out of the scope of the present study.

When analysing the transfusion outcome according to the different transplantation modalities within the three time periods, the best transfusion outcome of MRD HSCT fades during 2017–2020. Higher RBC and platelet PLT transfusion requirements have been reported in patients undergoing Haplo-HSCT, as compared to patients who receive a sibling donor peripheral blood stem cell graft [23,24]. In fact, our group showed that the blood product burden and cumulative incidence of RBC and PLT transfusion independence are similar for both UCBT and Haplo-HSCT modalities, respectively [25]. It has to be highlighted that during the last period in which PT-Cy was included, the transfusion burden and days to reach transfusion independence were quite similar for the different transplantation platforms.

Despite the higher transfusion burden as compared to previous periods, patients undergoing HSCT during the 2017–2020 period had significantly better overall survival. Multiple factors such as the use of different HSCT platforms, advances in supportive care in these time frames and PT-Cy-based GVHD prophylaxis could have contributed to this effect [26]. However, this analysis has not been performed in the present study.

Findings from previous studies have shown the benefits of reducing exposure to allogeneic blood in the HSCT setting [27] and the feasibility of the 1 RBC transfusion policy [28]. According to this trend, since 2017, we introduced the 1 RBC policy in the HSCT unit in order to optimize the use of blood products. However, we have not detected a reduction of transfusion needs. Again, the introduction of PT-Cy, among other changes, could have neutralized the positive effect of the 1-RBC transfusion strategy.

We are aware of the remarkable differences in the proportion of HSCT modalities and transfusion practices over time which would be very complex to analyse in detail. This is a limitation of the present study which mainly wants to offer an overview of transfusion management of HSCT over the years.

In conclusion, HSCT modalities have evolved and changed over time, while overall transfusion requirements have not significantly decreased and continue to be a cornerstone in transplantation-supportive care. More efforts to improve transfusion practices and implement patient blood management programs in the HSCT units may contribute to reducing the transfusion burden.

## Figures and Tables

**Table 1:** Patient characteristics according to different time periods.

Characteristics	2009–2012	2013–2016	2017–2020	*p*
Patients, n (%)	257 (30)	297 (35)	301 (35)	
Recipient age in years, median (range)	46 (16–66)	46.5 (16–70)	50.5 (15–71)	0.0007
Sex, n (%)				0.7
Male	151 (59)	168 (57)	178 (59)	
Female	106 (41)	129 (43)	123 (41)	
Diagnosis, n (%)				0.08
Acute myeloid leukaemia	115 (45)	131 (44)	140 (47)	
Acute lymphoblastic leukaemia	45 (17)	59 (20)	47 (16)	
Chronic lymphoproliferative disorders	38 (15)	54 (18)	37 (12)	
Multiple myeloma	8 (3)	11 (4)	22 (7)	
Myelodysplastic syndrome	16 (6)	23 (7)	22(7)	
Myeloproliferative disorders	12 (5)	7 (2)	17 (6)	
Chronic myeloid leukaemia	7 (3)	5 (1)	6 (2)	
Aplastic anaemia	13 (5)	7 (2)	9 (3)	
Others	3 (1)	0	1 (0)	
Donor type, n (%)				<0.0001
MRD	120 (47)	131 (44)	107 (35)	
MUD	14 (5)	26 (9)	94 (31)	
Haplo-HSCT	7 (3)	49 (16)	84 (29)	
UCBT	116 (45)	91 (31)	16 (5)	
Disease status at transplantation, n (%)				0.3
Early	118 (46)	151 (51)	150 (50)	
Intermediate	69 (27)	68 (23)	81 (27)	
Advanced	54 (21)	71 (24)	60 (20)	
Not applicable	16 (6)	7 (2)	10 (3)	
Stem cell source, n (%)				<0.0001
Bone marrow	2 (1)	4 (2)	26 (9)	
Peripheral blood	139 (45)	202 (68)	259 (86)	
Umbilical cord blood	116 (54)	91 (30)	16 (5)	
CD34+ × 10^6^/kg, median (range)	2.5 (0.04–14.4)	4.4 (0.08–15.5)	7.1 (0.1–25.9)	<0.0001
Conditioning regimen, n (%)				0.035
Myeloablative	146 (57)	163 (55)	141 (47)	
Reduced-intensity conditioning	111 (43)	134 (45)	160 (53)	
GVHD prophylaxis, n (%)				<0.0001
CsA + MTX	118 (46)	129 (43)	3 (1)	
CsA + corticosteroids	94 (37)	83 (28)	10 (3)	
CsA + MMF	37 (14)	28 (9)	11 (4)	
PT-Cy + CsA + MMF	1 (0)	45 (15)	15 (5)	
PT-Cy + MTX + sirolimus	0	0	259 (86)	
Other	7 (3)	12 (4)	3 (1)	
Transplant order, n (%)				
1	201 (78)	222 (75)	227 (75)	
2	55 (21)	70 (24)	66 (22)	
3	1 (0)	5 (1)	8 (3)	
ABO compatibility, n (%)				0.06
Identical	129 (50)	180 (61)	172 (57)	
Minor incompatibility	54 (21)	60 (20)	66 (22)	
Major incompatibility	62 (24)	45 (15)	45 (15)	
Bidirectional incompatibility	12 (5)	12 (4)	18 (6)	
Days until neutrophils > 0.5 × 10^9^/L, median (range)	14 (5–55)	14 (7–39)	17 (7–39)	<0.0001
Days until platelets > 20 × 10^9^/L, median (range)	23 (9–208)	20 (8–319)	28 (11–263)	<0.0001

MRD HLA-matched related donor; MUD HLA-matched unrelated donor; Haplo-HSCT, haploidentical hematopoietic stem cell transplant; UCBT, umbilical cord blood transplant; GVHD, graft versus host disease; CsA, cyclosporine A; MMF, mycophenolate mofetil; MTX, methotrexate; PT-Cy, posttransplant cyclophosphamide.

**Table 2:** RBC and PLT transfusion burden and transfusion independence of patients undergoing HSCT according to the 3 study periods. Cumulative incidence of competing events, Gray test and Kruskal–Wallis test were used to compare the groups.

	2009–2012	2013–2016	2017–2020	*p*
**MRD**				
Patients, n (%)	120 (34)	131 (36)	107 (30)	
RCB transfusion independence				**0.06**
Days, median (range)	10.5 (0–153)	11.5 (0–310)	17.5 (0–190)	
CuI at 100 days, % (95% CI)	85 (78–91)	85 (78–90)	79 (70–86)	
PLT transfusion independence				**0.0001**
Days, median (range)	11 (0–80)	11 (0–110)	19 (7–155)	
CuI at 100 days, % (95% CI)	94 (87–97)	94 (88–97)	87 (79–92)	
RBC units at day +30, median (range)	2 (0–21)	3 (0–24)	4 (0–38)	**<0.0001**
RBC units, day 31–90 *, median (range)	4 (2–53)	6 (2–49)	4 (1–30)	0.5
PLT units at day +30, median (range)	3 (0–55)	3 (0–70)	5 (1–39)	**0.002**
PLT units, day 31–90 *, median (range)	19 (1–99)	14 (1–83)	14 (1–85)	0.12
MUD				
Patients, n (%)	14 (11)	26 (19)	94 (70)	
RCB transfusion independence				0.61
Days, median (range)	25.5 (0–149)	12 (0–100)	26 (0–260)	
CuI at 100 days, % (95% CI)	78 (40–93)	80 (57–92)	84 (75–90)	
PLT transfusion independence				0.23
Days, median (range)	18 (6–22)	22 (6–173)	25 (3–210)	
CuI at 100 days, % (95% CI)	85 (53–96)	88 (68–96)	90 (81–95)	
RBC units at day +30, median (range)	5 (0–23)	4 (0–21)	4 (0–23)	0.31
RBC units, day 31–90 *, median (range)	4 (2–12)	5 (2–16)	5 (1–85)	0.8
PLT units at day +30, median (range)	10 (5–70)	6 (1–48)	7 (2–69)	0.12
PLT units, day 31–90 *, median (range)	6 (1–23)	2 (1–57)	10 (2–49)	0.07
Haploidentical				
Patients, n (%)	7 (5)	49 (35)	84 (60)	
RCB transfusion independence				0.097
Days, median (range)	29.5 (10–48)	25.5 (0–290)	21 (0–255)	
CuI at 100 days, % (95% CI)	28 (4–61)	67 (51–78)	74 (63–82)	
PLT transfusion independence				0.12
Days, median (range)	23 (6–35)	26 (22–72)	28 (0–169)	
CuI at 100 days, % (95% CI)	28 (4–61)	67 (52–78)	84 (73–90)	
RBC units at day +30, median (range)	10 (5–18)	6 (0–23)	5 (0–18)	0.08
RBC units, day 31–90 *, median (range)	2 (2–2)	6 (2–12)	6 (0–79)	0.15
PLT units at day +30, median (range)	14 (10–38)	11 (3–30)	8 (2–28)	0.13
PLT units, day 31–90 *, median (range)	11 (1–12)	10 (2–41)	10 (2–124)	0.3
UCBT				
Patients, n (%)	116 (52)	91 (41)	16 (7)	
RCB transfusion independence				0.17
Days, median (range)	33 (0–240)	28 (0–183)	33 (0–200)	
CuI at 100 days, % (95% CI)	63 (53–71)	54 (44–64)	73 (40–90)	
Platelet transfusion independence				**0.001**
Days, median (range)	35 (0–175)	34 (0–180)	34 (0–60)	
CuI at 100 days, % (95% CI)	73 (64–81)	67 (56–75)	93 (61–99)	
RBC units at day +30, median (range)	6 (0–18)	6 (0–39)	5 (3–12)	0.4
RBC units, day 31–90 *, median (range)	6 (3–29)	6 (3–53)	6 (3–12)	0.8
PLT units at day +30, median (range)	16 (0–59)	15 (0–85)	11 (0–30)	0.19
PLT units, day 31–90 *, median (range)	8 (1–98)	6 (1–300)	4 (1–50)	0.48

* among transfused patients. MRD HLA-matched related donor; MUD HLA-matched unrelated donor; UCBT, umbilical cord blood transplant; CuI, cumulative incidence; CI, confidence interval; RBC, red blood cell; PLT, platelet. Bold font indicates statistical significance

## Data Availability

The anonymized data presented in this study are available at the request of the author. The data are not publicly available due to current personal data protection legislation.
